# Targeted Knock-Down of miR21 Primary Transcripts Using snoMEN Vectors Induces Apoptosis in Human Cancer Cell Lines

**DOI:** 10.1371/journal.pone.0138668

**Published:** 2015-09-25

**Authors:** Motoharu Ono, Kayo Yamada, Fabio Avolio, Vackar Afzal, Dalila Bensaddek, Angus I. Lamond

**Affiliations:** Centre for Gene Regulation and Expression, School of Life Sciences, University of Dundee, Dundee, United Kingdom; CNRS UMR7622 & University Paris 6 Pierre-et-Marie-Curie, FRANCE

## Abstract

We have previously reported an antisense technology, ‘snoMEN vectors’, for targeted knock-down of protein coding mRNAs using human snoRNAs manipulated to contain short regions of sequence complementarity with the mRNA target. Here we characterise the use of snoMEN vectors to target the knock-down of micro RNA primary transcripts. We document the specific knock-down of miR21 in HeLa cells using plasmid vectors expressing miR21-targeted snoMEN RNAs and show this induces apoptosis. Knock-down is dependent on the presence of complementary sequences in the snoMEN vector and the induction of apoptosis can be suppressed by over-expression of miR21. Furthermore, we have also developed lentiviral vectors for delivery of snoMEN RNAs and show this increases the efficiency of vector transduction in many human cell lines that are difficult to transfect with plasmid vectors. Transduction of lentiviral vectors expressing snoMEN targeted to pri-miR21 induces apoptosis in human lung adenocarcinoma cells, which express high levels of miR21, but not in human primary cells. We show that snoMEN-mediated suppression of miRNA expression is prevented by siRNA knock-down of Ago2, but not by knock-down of Ago1 or Upf1. snoMEN RNAs colocalise with Ago2 in cell nuclei and nucleoli and can be co-immunoprecipitated from nuclear extracts by antibodies specific for Ago2.

## Introduction

snoMEN (snoRNA Modulator of gene ExpressioN) vectors provide a form of antisense technology for modulating the expression of target genes based upon complementary base pairing interactions, analogous to the more familiar siRNA/shRNA vector systems[1]. The snoMEN vector technology is created by manipulation of the human box C/D small nucleolar RNA (snoRNA) HBII-180C. This class of snoRNAs contain an internal sequence (M box) that can be altered to make it complementary to RNA targets. snoRNAs are a family of conserved nuclear RNAs concentrated in nucleoli where they either function in the modification of ribosomal RNA (rRNA), or participate in the processing of rRNA during ribosome subunit synthesis[2–5]. Box C/D snoRNAs are named after a common RNA motif in this subfamily that serves as a binding site for a group of box C/D proteins, including NOP56, NOP58, 15.5K and the highly conserved protein fibrillarin, which has the specific 2’-O-methylase activity. Most snoRNAs are encoded within intron sequences, either located in the primary transcripts of protein coding genes, or in dedicated transcripts containing tandem arrays of multiple snoRNAs. Endogenous snoRNAs are highly abundant nuclear RNAs that are efficiently processed from primary transcripts. Thus, processing and delivery of snoMEN RNAs is similarly efficient and not prone to saturation of the host cell processing machinery when snoMEN are expressed from exogenous vectors.

In previous studies it was shown that snoMEN vectors can reduce protein expression levels by knocking-down the expression of nuclear pre-mRNAs, allowing the targeting of complementary sequences within intron and/or non-coding 5’ and 3’ flanking sequences within mRNA precursors (pre-mRNAs)[6]. This effectively increases the range of sequences in target RNAs that can be explored to achieve gene-specific inhibitory effects. In common with endogenous snoRNAs, snoMEN RNAs are efficiently transcribed from RNA polymerase II promoters, rather than from the RNA polymerase III promoters used for shRNA plasmids[7]. As snoMEN RNAs are encoded within introns, it is relatively easy to design vectors that can express multiple snoMEN within different introns of a single transcript, which also encodes a protein reporter. This facilitates the creation of either transient, or stable, gene knock-ins, accomplished using a single transcript, driven from a single promoter. This approach using snoMEN vectors has thus been used to establish human ‘protein replacement’ stable cell lines, where expression of a targeted protein is reduced by snoMEN RNAs and effectively substituted by the expression of a recombinant protein encoded by the same transcript used to deliver the snoMEN[6].

Cancer and other proliferative diseases (such as auto-immune disease and inflammation) are frequently associated with abnormal apoptosis, or cell death. In cancer cells, for example, the mechanisms are usually disrupted that induce programmed cell death following either serious DNA damage and/or defects in normal cell cycle progression, thereby allowing cancer cells to avoid apoptosis. A potential approach to cancer therapy is thus to trigger apoptosis by finding a way to overcome the mechanisms that are blocking the endogenous signalling pathways that would otherwise lead to death of the cancer cells. It is now thought that one of the contributing mechanisms allowing many forms of cancer cells to suppress activation of cell death pathways is mediated by overexpression of specific microRNAs, such as miR21[8].

miR21 was one of the first miRNAs detected in the human genome and displays strong evolutionary conservation across a wide range of vertebrate species, including mammalian, avian and fish clades[9]. RNA expression profiles, detected using high-throughput transcriptome profiling approaches, which compare miRNAs in tumours and other cell lines associated with cancer with those of normal cells/tissues, strikingly suggest that miR21 is over expressed in the vast majority of cancer types analysed[8]. More recently, antisense studies targeting mature miR21 suggested that blocking miR21 function can induce apoptosis by activating expression of the programmed cell death 4 (PDCD4) protein in certain types of cancer cells, e.g. HeLa cells and MCF-7 cells[10, 11]. The inhibition of miR21 was reported using synthetic antisense oligonucleotide analogues, complementary to miR21[12, 13]. However, while miR21 knock-down via antisense modified oligonucleotides was reported to arrest tumour cell proliferation, both *in vitro* and *in vivo*, issues remain in using this approach for clinical therapy, relating to the low efficiency of the delivery system and to the potential side-effects of antisense oligonucleotides that still have to be addressed. The use of siRNA/shRNA as an alternative mechanism for targeting inhibition of miRNAs has been suggested as a therapeutic, but also includes potential risks, such as off-target effects and disruption of the RNA-induced silencing complex (RISC) in the cell[14–17]. Nonetheless, several studies have described inhibiting expression of mature miR21 using siRNA/shRNA, specifically affecting only the 22 base processed miRNA but not causing knock-down of the miR21 primary transcript[18].

Micro RNAs (miRNAs) comprise a family of short, regulatory RNAs, typically 22 nucleotides in length, which can post-transcriptionally regulate gene expression *in vivo*. In mammals, miRNAs have been reported to regulate gene expression mainly by mechanisms that inhibit translation of protein coding transcripts, likely through base-pairing to specific target sequences in the mRNA 3’ untranslated regions (UTRs)[19]. In some cases miRNAs are transcribed as an independent transcription unit that does not code for protein. However, many miRNAs are located within the intron sequences of protein-coding genes. These intron-encoded miRNAs are thus co-expressed with their host protein coding genes[20–25]. Mature miRNAs are processed from longer pre-miRNA transcripts, which are usually ~70 nucleotide-long, hairpin structures. These pre-miRNAs in turn are processed from the original primary gene transcripts, often referred to as pri-miRNAs[26].

In this study, we show that snoMEN vectors can be used to knock-down miR21 by targeting specific sequences within pri-miRNA transcripts, resulting in apoptosis of human cancer cell lines. We investigate factors required for snoMEN mediated inhibition of miRNA expression and show that snoMEN can be delivered in cells from both plasmid and lentiviral vectors.

## Results

### snoMEN targeting pri-miR21 induce apoptosis in transformed cells

**Fig 1:**
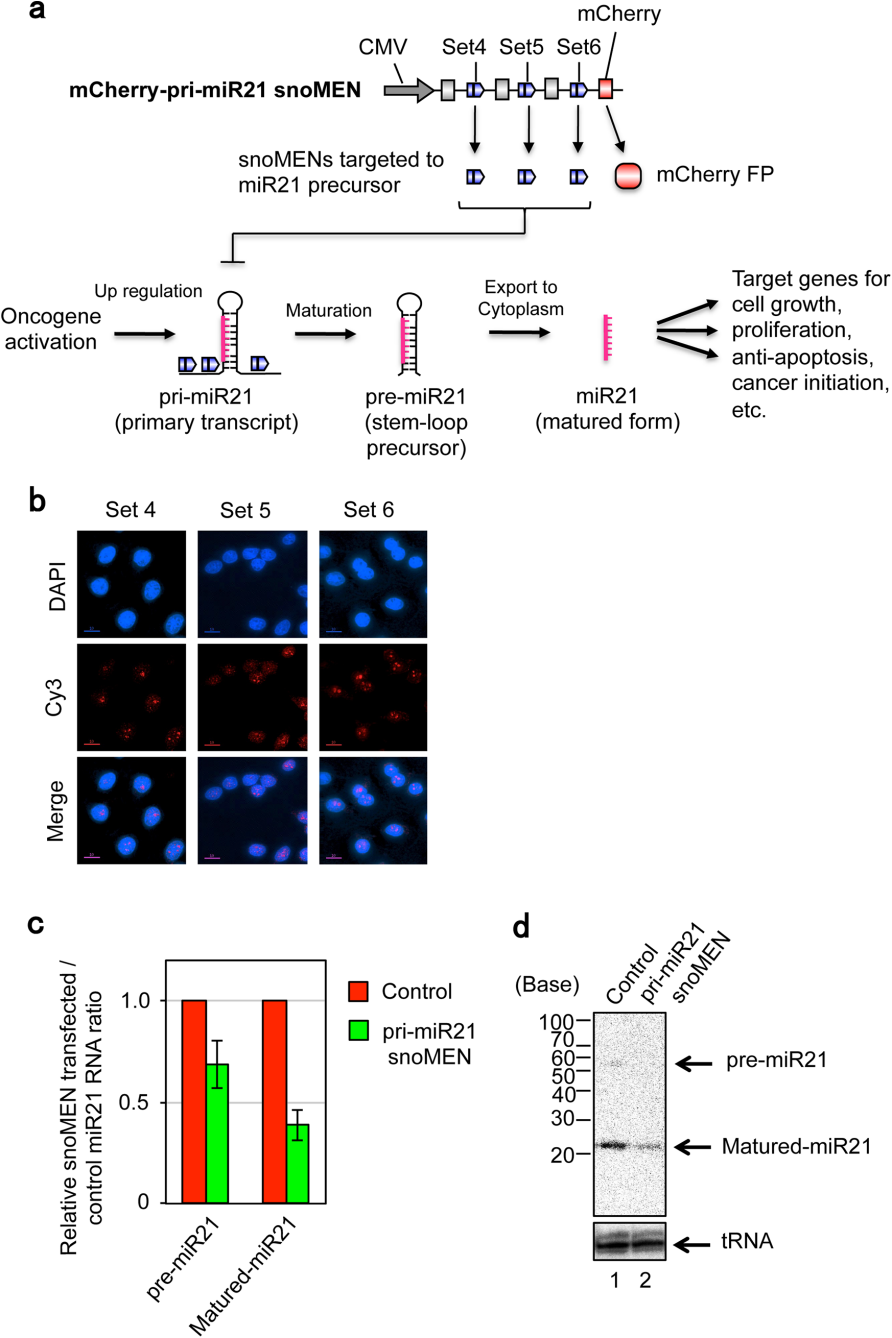
snoMEN vector targeted to miR21 primary transcript. (**a**) Structure for targeted endogenous miR21 primary transcript (mCherry–pri-miR21 snoMEN) and schematic diagram of miR21 maturation pathway. This construct has three snoMEN RNAs (blue pentagons) as previously described[1], except that here the M box sequences are complementary to specific sequences within the endogenous miR21 primary transcript. (**b**) Validation of snoMEN expression by Fluorescence In Situ Hybridisation (FISH) analysis. Each snoMEN RNA was detected by using a M box specific RNA probe labelled with Cy3 (Cy3). DNA is stained by DAPI (DAPI). Scale bar is 10 μm. (**c**) RNA analysis. Total RNA from HeLa cells was harvested 24 hours after transfection and qRT-PCR was performed to identify miR21 precursor molecules using miR21 precursor specific primers (pre-miR21). Following cDNA synthesis, qPCR was performed using matured miR21 specific primer and universal primer provided by PerfeCta SYBR Green qPCR kit (Quanta Biosciences, see also methods) (Matured-miR21). U3 was used as a loading control. Graph depicts mean and standard deviation from a minimum of 5 independent experiments. (**d**) HeLa cells transfected with pri-miR21-snoMEN/Control for 24 hours prior to total RNA extraction and northern blot analysis.

Our hypothesis is that if snoMEN can be targeted to cause a reduction in the levels of the miR21 primary transcript this may induce apoptosis in tumour cells whose survival is dependent upon high levels of miR21 expression. Therefore, to test whether snoMEN can be used to modify the expression of miR21, a plasmid vector expressing M box-modified snoRNAs targeted to endogenous pri-miR21 was constructed (**Fig 1A**), based on the previous snoMEN design[1]. This snoMEN vector (mCherry–pri-miR21 snoMEN) encoded three snoMEN, which each targeted different regions of the pri-miR21 sequence. These three snoMEN RNAs are each encoded within separate introns of the same RNA pol II transcript, which also encodes the mCherry protein that acts as a fluorescent protein (FP) marker in transfected cells. Following transfection of the mCherry–pri-miR21 snoMEN plasmid vector into HeLa cells, FISH (fluorescence *in situ* hybridisation) experiments, using snoMEN-specific probes, showed a steady state localisation pattern for the snoMEN RNAs that was predominantly nuclear, with accumulation in nucleoli (**Fig 1B** and other data not shown). This pattern is consistent with previous snoMEN expression studies[1, 6].

**Fig 2:**
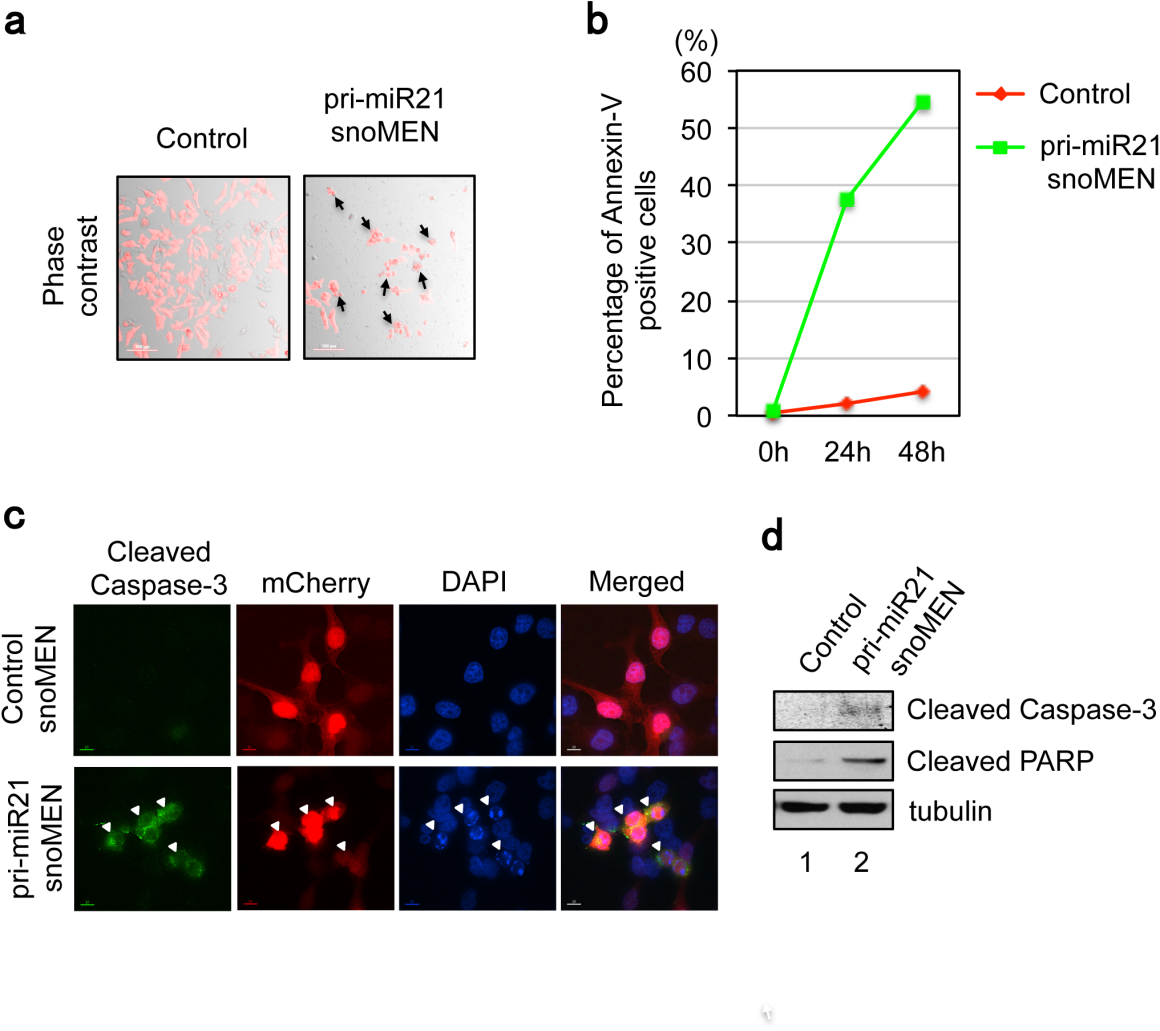
miR21 knock-down by snoMEN vector resulted in apoptosis phenotype. (**a**) Micrographs showing examples of apoptosis induction in HeLa cells transfected with pri-miR21-snoMEN. Images were taken 72 hours after transfection. Arrows indicate the cells showing an apoptosis phenotype. Scale bar is 10 μm. (**b**) Graph shows a time course of changes in the population of Annexin-V (apoptosis marker) positive cells after transfection with either pri-miR21-snoMEN (Green), or Control (Red). The cell number was counted using FACS. Annexin-V signal was detected using Guava Nexin Reagent (Guava technologies). (**c**) Images show localisation pattern of DNA (DAPI, Blue), a transfection FP-marker of either pri-miR21-snoMEN/Control (mCherry, Red) and an Apoptosis marker (Cleaved Caspase-3, Green). Arrowheads show Apoptosis marker positive cells. Scale bar is 10 μm. (**d**) Western blot showing maker proteins in HeLa cells transfected with pri-miR21-snoMEN/Control for 24 hours prior to protein extraction. Whole cell lysates were analysed using the indicated antibodies.

**Fig 3:**
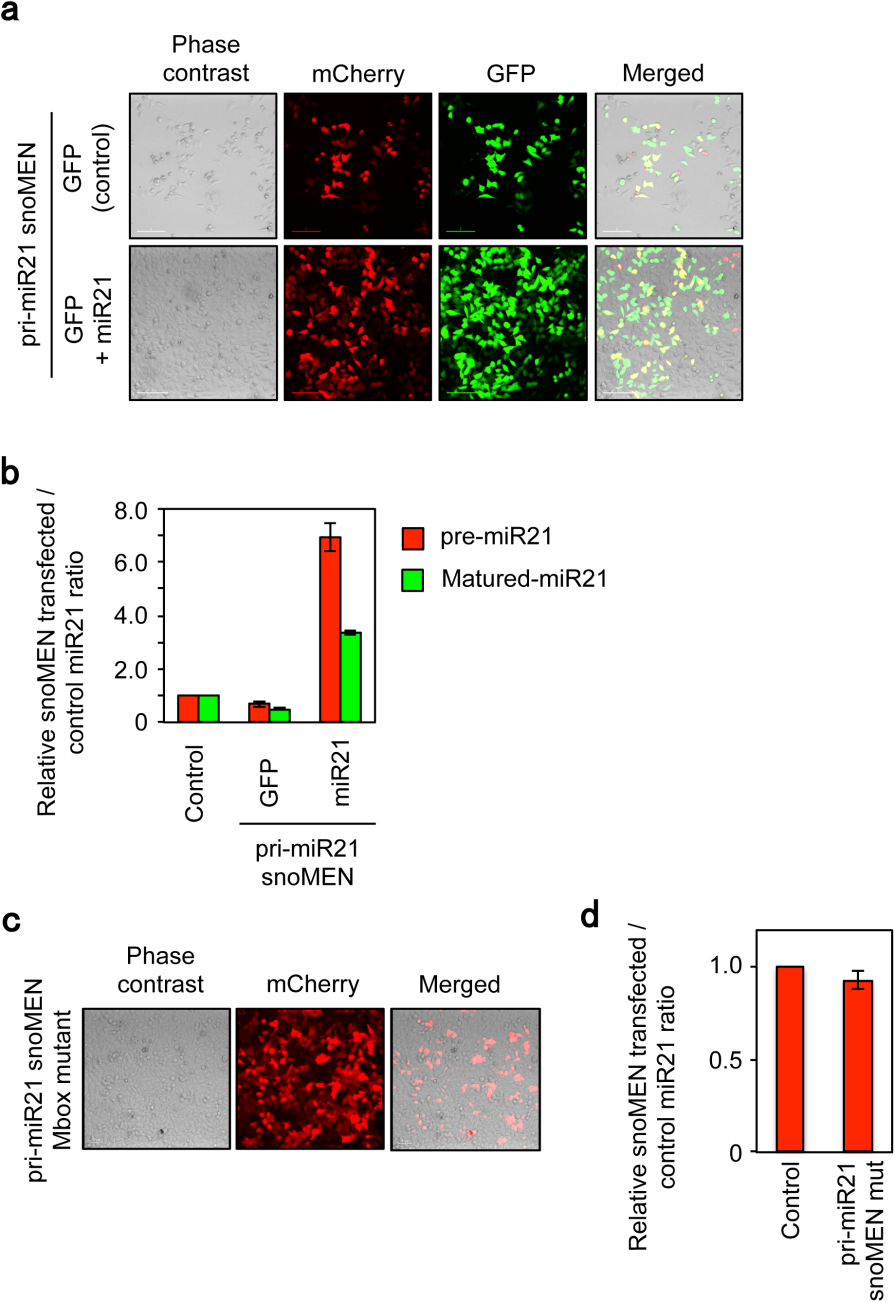
Compensatory analysis of miR21 knock-down targeted to miR21 primary transcript by snoMEN vector. (**a**) Micrographs showing rescue experiment of apoptosis induction by co-transfecting with pri-miR21-snoMEN (mCherry) and pre-miR21 expression plasmid (GFP+miR21)/Control plasmid that expresses GFP protein alone (GFP). Images were taken 72 hours followed after transfection. Scale bar is 10 μm. (**b**) RNA analysis. Total RNA from HeLa cells was harvested 24 hours after co-transfection with pri-miR21-snoMEN (mCherry) and pre-miR21 expression plasmid (miR21)/Control plasmid that express GFP protein alone (GFP) and qRT-PCR was performed to identify miR21 precursor molecules using miR21 precursor specific primers (pre-miR21, Red). Following cDNA synthesis, qPCR was performed using matured miR21 specific primer and universal primer provided by the PerfeCta SYBR Green qPCR kit (Quanta Biosciences, see also methods) (Matured-miR21, Green). U3 snoRNA was used as a control. Graph depicts mean and standard deviation from a minimum of 3 independent experiments. (**c**) Micrographs show prevention of non-apoptosis induction by transfecting with pri-miR21-snoMEN mut, which has 3 base mismatches in each M box complementary sequence to pri-miR21. Images were taken 72 hours followed after transfection. Scale bar is 10 μm. (**d**) RNA analysis. Total RNA from HeLa cells was harvested 24 hours after transfection with either pri-miR21-snoMEN mut or Control. Following cDNA synthesis, qPCR was performed using matured miR21 specific primer and universal primer provided by the PerfeCta SYBR Green qPCR kit (Quanta Biosciences, see also methods). U3 snoRNA was used as a control. Graph depicts mean and standard deviation from a minimum of 4 independent experiments.

At 24 hours after transient transfection of the snoMEN plasmid mCherry–pri-miR21 into HeLa cells, which highly express miR21[27], we observed ~65% and ~45% knock-down for pre-miR21 and matured miR21, respectively, compared with a negative control snoMEN (Control) vector, as judged by both qRT-PCR (**Fig 1C**) and northern blotting analysis (**Fig 1D**). Furthermore, cells transfected with the mCherry–pri-miR21 snoMEN plasmid showed high levels of apoptosis (**Fig 2A** arrow). In contrast, cells transfected with a control snoMEN plasmid (Control), which doesn’t target any endogenous genes, did not show signs of apoptosis (**Fig 2A**). Multiple markers of apoptosis were tested in each case, including an Annexin-V assay using FACS (Fluorescence-activated cell sorting) (**Fig 2B**), an immunofluorescence analysis of cleaved Caspase-3 (**Fig 2C**), Western blotting analysis for cleaved PARP1 and cleaved Caspase-3 (**Fig 2D**). All of these assays showed up-regulation of apoptosis specifically in cells transfected with the mCherry–pri-miR21 snoMEN plasmid. However, co-transfection of plasmids miR21 shRNA–pLVX (which expresses pre-miR21) and mCherry–pri-miR21 snoMEN, resulted in little or no cytotoxicity and apoptosis, compared with co-transfection of cells with mCherry–pri-miR21 snoMEN and the negative control EGFP–pLVX (expressing EGFP protein alone) expression plasmids (**Fig 3A and 3B**). These results indicate that the apoptosis caused by snoMEN-mediated miR21 depletion can be rescued by exogenous miR21 over expression.

To investigate the relationship between the sequence in the M box region of the snoMEN and the pri-miR21 target sequence, a mutant version of mCherry–pri-miR21 snoMEN plasmid (pri-miR21 snoMEN mut), which included three mismatches in the pri-miR21 complementary sequence, was made and assayed to measure both knock-down levels and any potential apoptosis phenotype (**Fig 3C and 3D**). The results show that the 3 base mismatch mutations in the M box effectively eliminate both the apoptosis phenotype and knock-down activity, comparable with the negative control snoMEN vector (**Fig 3C and 3D**). We conclude that miR21 knock-down and the resulting apoptosis phenotype seen with the mCherry–pri-miR21 snoMEN vector is dependent upon sequence complementarity between the M box region and the targeted region of pri-miR21.

We also constructed and tested another snoMEN vector, mCherry–pre-miR21 snoMEN, which targets miR21 precursor sequences (**Fig A** in S1 File). Transient transfection of the mCherry–pre-miR21 snoMEN plasmid into HeLa cells resulted in miR21 knock-down and induction of apoptosis, similar to the results obtained above with the mCherry–pri-miR21 snoMEN plasmid. The results for mCherry–pre-miR21 snoMEN and mCherry–pri-miR21 snoMEN plasmids were similar with respect to FISH analysis, the Annexin-V assay using FACS and miR21 knock-down levels, as analysed by qRT-PCR, (**Figs B-D** in S1 File). These results indicate that snoMEN vectors can indeed target miRNA primary transcripts, which provides more flexibility in the design of knock-down vectors than targeting only short (~22 base), mature miRNA sequences.

**Fig 4:**
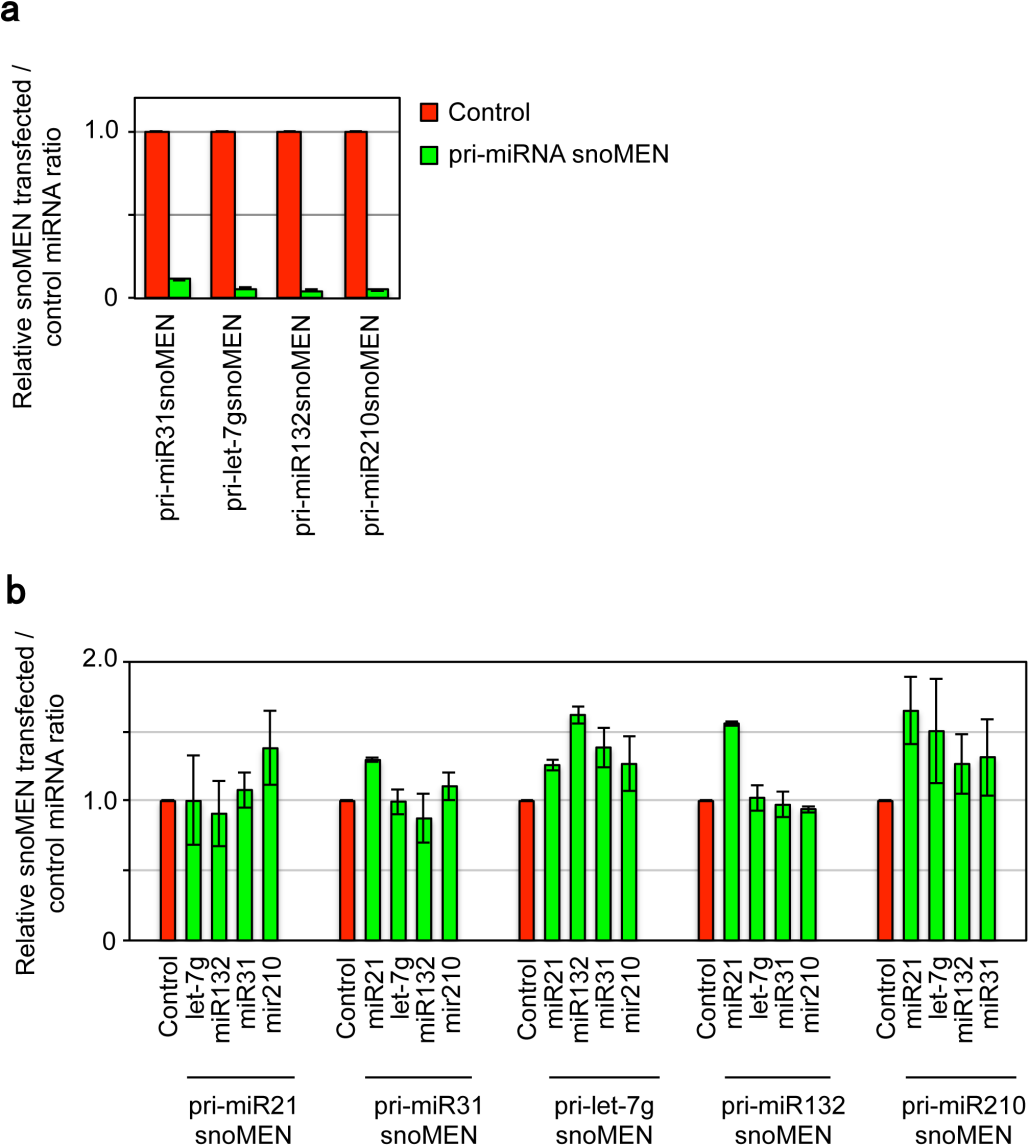
Specificity of miRNA knock-down using snoMEN vectors. (**a**) snoMEN vectors targeted to additional miRNAs. Total RNA from HeLa cells was harvested 24 hours after transfection and following cDNA synthesis, qPCR was performed using matured miRNAs targeted specific primers, i.e. miR31, let-7g, miR132, miR210, and universal primer provided by the PerfeCta SYBR Green qPCR kit (Quanta Biosciences, see also methods) (Matured-miRNA, Green). U3 snoRNA was used as a control. Graph depicts mean and standard deviation from a minimum of 3 independent experiments. (**b**) Analysis of specificity of targeting miRNA knock-down using snoMEN vectors. qPCR was performed for 5 different miRNAs (miR21, miR-31, let-7g, miR132, miR-210) to check possible off target effects of snoMEN vectors targeting pri-miRNAs, i.e. pri-miR21 snoMEN, pri-miR31 snoMEN, pri-let-7g snoMEN, pri-miR132 snoMEN, pri-miR210 snoMEN. The graph shows average signal ratio for three independent experiments comparing specific targeted snoMEN with Control snoMEN transfection (Control, Red). Each signal was normalised by comparison with U3 snoRNA level.

Next, we tested whether snoMEN vectors could be used to target other miRNAs, distinct from miR21. Thus, snoMEN vectors were designed to target four previously well characterised miRNA primary transcripts [27–30], i.e., pri-miR31, pri-let-7g, pri-miR132 and pri-miR210, and their knock-down activity measured using qRT-PCR following transient transfection into HeLa cells (**Fig 4A**). This showed specific and reproducible knock-down for all four targeted miRNAs, compared with the negative control snoMEN, as judged by qRT-PCR. Furthermore, for each of the targeted miRNAs in this study we investigated the specificity of miRNA knock-down by the snoMEN vectors. To do this we used qRT-PCR to measure and compare the levels of the respective targeted and non-targeted micro RNAs upon expression of either a miRNA targeted snoMEN RNA, or the negative control snoMEN RNA (**Fig 4B**). This showed, for example, that expression of the mCherry–pri-miR21 snoMEN caused a specific reduction in miR21 levels with little or no off-target effects on the levels of the other four micro RNAs tested. Similar results were obtained for each of the snoMEN vectors targeted to the four other miRNAs tested above, i.e., pri-miR31, pri-let-7g, pri-miR132 and pri-miR210.

### Expression of snoMEN from a lentiviral vector

**Fig 5:**
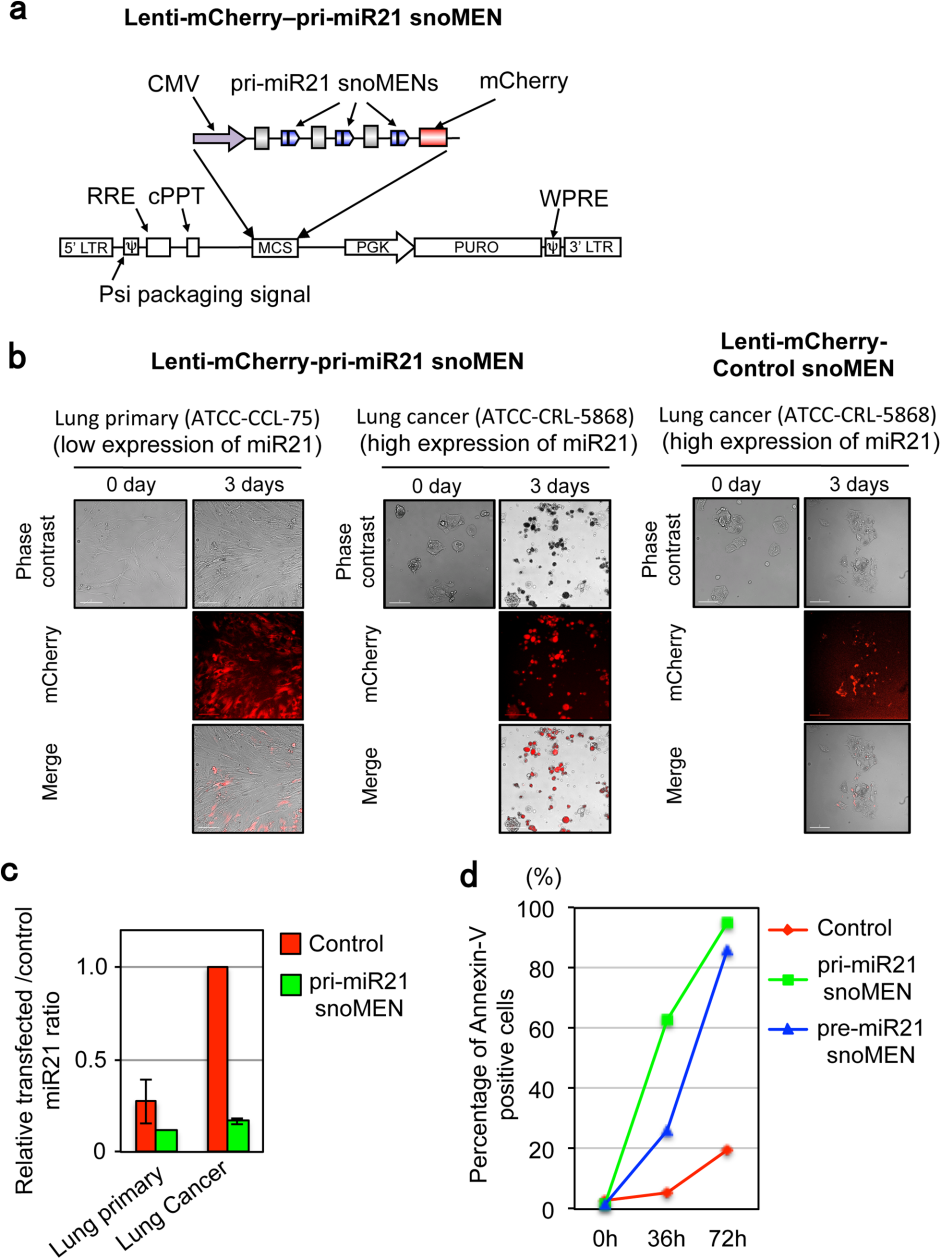
snoMEN knock-down delivered using a lentiviral vector. (**a**) Structure of lentiviral vector encoding snoMEN targeted to the endogenous miR21 primary transcript (Lenti-mCherry–pri-miR21 snoMEN). This construct has three snoMEN RNAs (blue pentagons) and an mCherry FP-marker cDNA as described in **Fig 1A**, except that the insert was sub-cloned into a vector that would produce the lentivirus particle (pLVX-puro, Clontech). (**b**) Micrographs show apoptosis induction by transducing with either Lenti-mCherry–pri-miR21-snoMEN, or Lenti-mCherry–Control-snoMEN virus particles. Images were taken 72 hours after transduction. Scale bar is 10 μm. (**c**) Total RNA from human lung primary (ATCC-CCL-75) and lung Cancer (ATCC-CRL-5868) cells was harvested 24 hours after transduction. Following cDNA synthesis, qPCR was performed using both a matured miR21 specific primer and a universal primer provided by the PerfeCta SYBR Green qPCR kit (Quanta Biosciences, see also methods). GAPDH was used as a control. Graph depicts mean and standard deviation from a minimum of 4 independent experiments. (**d**) Graph shows a time course of changes in the population of Annexin-V positive cells transduced with either lenti-pri-miR21-snoMEN (Green), lenti-pre-miR21-snoMEN (Blue) or Control (Red). The cell number was counted using FACS. The Annexin-V signal was detected using Guava Nexin Reagent (Guava technologies).

To improve the flexibility of delivering snoMEN into cells, in particular aiming to improve the delivery of snoMEN in cell lines that show low transfection efficiencies for plasmid vectors, we sought to construct lentiviral vectors that can express snoMEN (**Fig 5A**). Most mammalian cells are susceptible to lentivirus infection, including both dividing and non-dividing cells, stem cells, and primary cells[31]. We compared lentiviral expression of pri-miR21 snoMEN in either primary, or cancer cells, derived from human tissues. Two separate lentiviral snoMEN vectors were constructed, i.e., lenti-mCherry–pri-miR21 snoMEN and lenti-mCherry–pre-miR21 snoMEN, which target different regions of the miR21 primary transcript (**Fig 5A** and also see **Fig A** in S1 File). Both pri-miR21 snoMEN & pre-miR21 snoMEN also encode mCherry cDNA as an expression marker for transfected cells. As shown by FISH analysis, each of the lenti-mCherry–pri-miR21 snoMEN, lenti-mCherry–pre-miR21 snoMEN and negative control lenti-mCherry–Control snoMEN (EGFP targeted), express snoMEN RNAs that accumulate in nucleoli, similar to the results obtained when snoMEN RNAs are expressed from transfected plasmid vectors (**Fig E** in S1 File and **Fig 1B**)[1]. The expression of snoMEN RNAs from the viral vectors was also confirmed by northern blot analysis (Data not shown).

Next, lenti-mCherry-pri-miR21 viruses were transduced either into WI-38 (ATCC-CCL-75) human lung fibroblast cells (isolated from a foetus at 3 months gestation), which express low levels of miR21, or into human lung adenocarcinoma cells, established from a human patient (age 55, stage 2), which highly express miR21 (**Fig F** in S1 File). This miR21 expression level didn’t change after transducing a lenti-mCherry–Control snoMEN vector (**Fig F** in S1 File). These cells are difficult to transfect with DNA plasmids using general transfection reagents. More than 90% of both primary and cancer cells showed mCherry expression 24 hours after lentivirus transduction. Primary cells transduced by either pri-miR21 snoMEN, or pre-miR21 snoMEN, kept growing and continued to express mCherry (**Fig 5B** and **Fig G** left panels in S1 File). However, the lentivirus transduced lung adenocarcinoma cells showed a strong cytotoxic phenotype within 3 days of transduction (**Fig 5B** middle panel and **Fig G** right panels in S1 File). While this cancer cell specific cytotoxic phenotype was observed following transduction with either the lenti-mCherry–pri-miR21 snoMEN, or the lenti-mCherry–pre-miR21 snoMEN vector, transduction with the negative control lentiviral vector expressing snoMEN targeting EGFP (lenti-mCherry–Control snoMEN; no endogenous target), didn’t show a cytotoxic phenotype in either the primary, or lung adenocarcinoma cells (**Fig 5B** right panel).

Transduction of either lenti-mCherry–pri-miR21 snoMEN, or lenti-mCherry–pre-miR21 snoMEN, into lung adenocarcinoma cells, which highly express miR21, resulted in each case in ~25% reduction in the levels of mature miR21 RNA, compared with cells transduced with the negative control, lenti-mCherry–Control snoMEN vector, as judged by qRT-PCR (**Fig 5C** and **Fig H** in S1 File). Furthermore, the Annexin-V assay, using FACS, showed up-regulation of Annexin-V in the lung adenocarcinoma cells following transduction with either lenti-mCherry–pri-miR21 snoMEN, or lenti-mCherry–pre-miR21 snoMEN, but not after transduction with the negative control lentiviral snoMEN vector (**Fig 5D**).

We conclude that lentiviral vectors able to express snoMEN RNAs can be transduced into mammalian cells with high efficiency. Delivery of snoMEN targeted to sequences in pri-miR21 induces apoptosis in human lung adenocarcinoma cells but not in untransformed human lung primary cells.

### Comparison of siRNA/shRNA and snoMEN pri-miRNA interference

**Fig 6:**
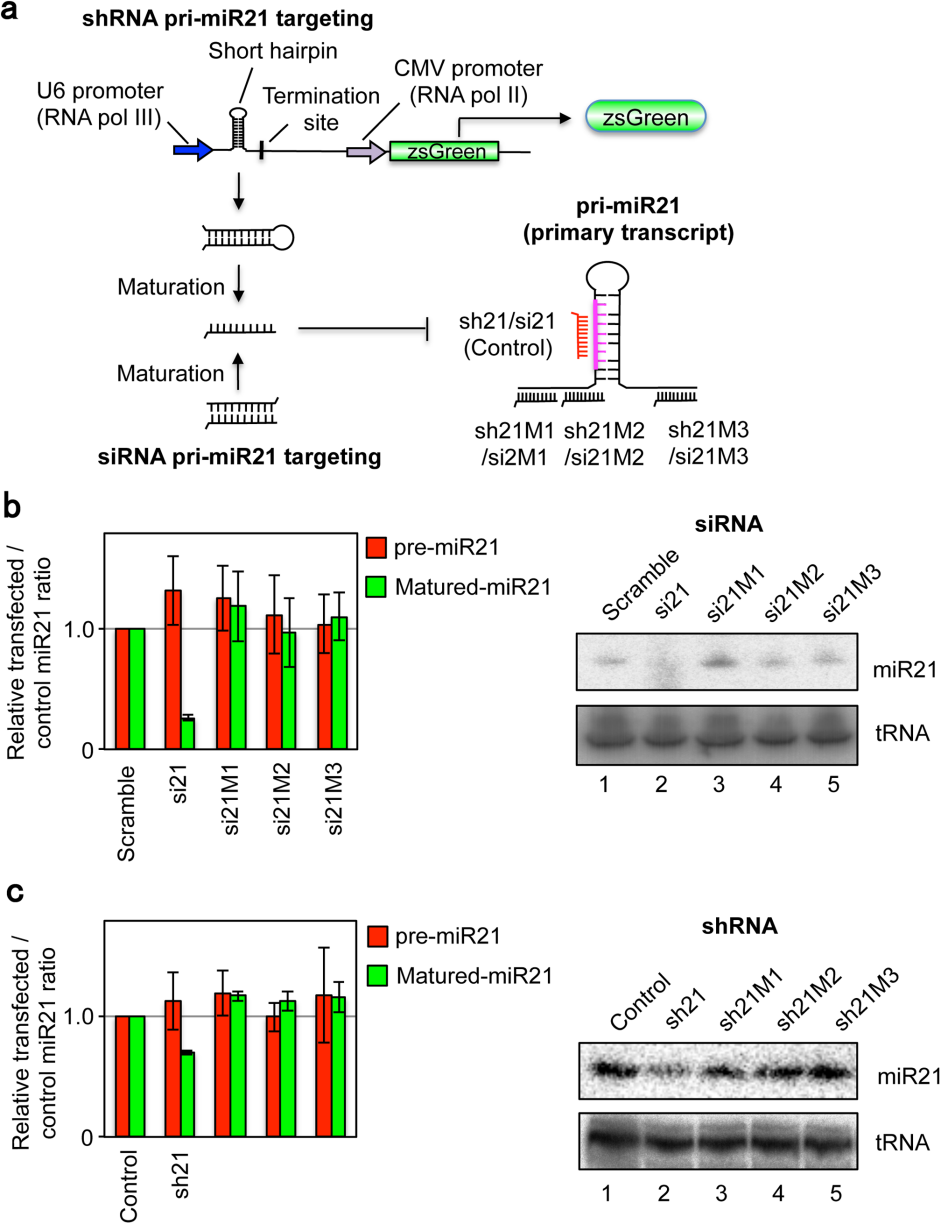
miR21 knock-down using si/shRNA targeted to endogenous miR21 primary transcript. (**a**) The regions on the pri-miR21 RNA targeted by the snoMEN vector used in this study are shown in a schematic diagram (sh/si21M1-M3). The same pri-miR21 sequences as targeted by the snoMEN vector were targeted by siRNA oligoribonucleotides and shRNA expression plasmids. (**b**) The graph shows the result of qRT-PCR/qPCR. Total RNA from HeLa cells was harvested 24 hours after transfection and qRT-PCR was performed to identify miR21 precursor molecules using miR21 precursor specific primers (pre-miR21, Red). Following cDNA synthesis, qPCR was performed using matured miR21 specific primer and universal primer provided by the PerfeCta SYBR Green qPCR kit (Quanta Biosciences, see also methods) (Matured-miR21, Green). U3 was used as a control. Graph depicts mean and standard deviation from a minimum of 4 independent experiments. The right panel shows the result of Northern blot analysis for siRNA experiments. Detection of endogenous miR21 RNA levels following transfection of HeLa cells using either control siRNA (Scramble: lane1), miR21 siRNA (si21: lane2), miR21 M box siRNA-1 (si21M1: lane3), miR21 M box siRNA-2 (si21M2: lane4), miR21 M box siRNA-3 (si21M3: lane5). An equivalent amount of HeLa total RNA was loaded for each lane and the RNA separated by PAGE, electroblotted onto membrane and probed both with a miR21 probe and with a tRNA probe as a loading control. (**c**) The same series of experiments with siRNA transfection, as described in **Fig 6b**, except shRNA expression plasmids were transfected instead of siRNAs.

Having shown above that M box-modified snoRNAs can reduce expression of endogenous miRNAs in human cells when targeted to a sequence within the primary transcript of a miRNA that is not present in the mature miRNA, we next compared the ability of siRNA oligoribonucleotides to knock-down expression of miR21 when targeted against the same primary transcript sequences (**Fig 6A**). To perform these comparisons, three siRNA oligoribonucleotides complementary to the same primary transcript sequences in miR21 pri-miRNA as targeted by the M box-modified snoRNAs in mCherry–pri-miR-21 snoMEN, were transfected into HeLa cells (**Fig 6A** and **Fig I** in S1 File). All three pri-miRNA siRNAs (si21M1–3), showed little or no knock-down of either mature-miR21 or pre-miR21 levels, as did a further negative control siRNA (Scramble), as judged by qRT-PCR (**Fig 6B** left panel) and by Northern blotting (**Fig 6B** right panel). As a positive control, another siRNA targeted to the mature miR21 sequence (si21), resulted in ~25% knock-down, specifically for mature miR21 but not for pre-miR21, consistent with previous reports[12, 13]. Furthermore, the same analyses were performed also using shRNA technology (**Fig 6A and 6C** and **Fig I** in S1 File). Thus, three shRNA expression plasmids per gene, complementary to the same primary transcript sequences in miR21 as targeted by the M box-modified snoRNAs in mCherry–pri-miR21 snoMEN, were transfected into HeLa cells (**Fig 6A** and **Fig I** in S1 File). All three pri-miR21 targeted shRNAs (sh21M1–3), again showed little or no knock-down in the levels of either mature-miR21, or pre-miR21, as did a further negative shRNA control, as judged by qRT-PCR (**Fig 6C** left panel) and by Northern blotting (**Fig 6C** right panel). In contrast, a positive control shRNA plasmid expressing an shRNA targeted to the mature miR21 sequence (sh21), resulted in ~70% knock-down of mature miR21.

We conclude that the snoMEN vectors can cause a reduction in the expression of specific miRNAs by targeting nuclear precursor RNA sequences that do not appear to be amenable to knock-down by either siRNA oligoribonucleotides, or shRNA vectors. In contrast, cytoplasmic RNAs, including mature miRNAs, can be knocked-down by shRNA vectors that target the same sequences used in the snoMEN vectors.

### Mechanism of snoMEN RNA interference

**Fig 7:**
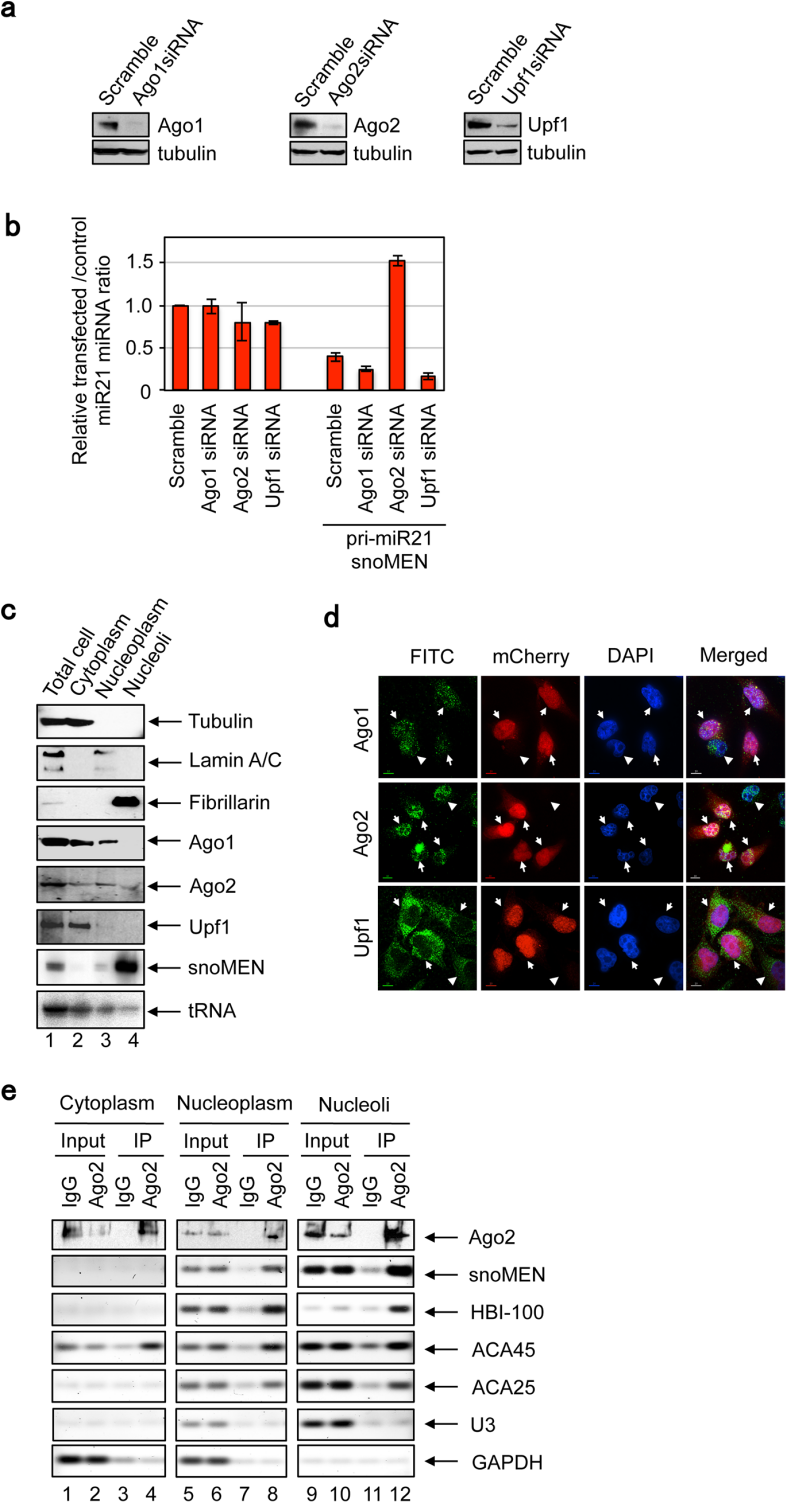
Ago2 is important for snoMEN RNA interference. (**a**) HeLa cells were transfected with siRNA oligonucleotides for Ago1, Ago2, and Upf1. Whole cell lysates were prepared 48 hours after transfections and analysed by western blot using the indicated antibodies. (**b**) HeLa cells were transfected with siRNA oligonucleotides prior to pri-miR21-snoMEN transfection for 24h. Total RNA from HeLa cells was harvested and qPCR was performed, followed by cDNA synthesis, using matured miR21 specific primer and universal primer provided by the PerfeCta SYBR Green qPCR kit (Quanta Biosciences, see also methods). (**c**) HeLa cells transfected with pri-miR21-snoMEN for 24 hours prior to protein extraction. The same amount of proteins/RNAs from each subcellular fraction was loaded on each lane and analysed by western blot/Northern blot using the indicated antibodies (Ago1, Ago2, Upf1) / RI-labelled probes (snoMEN, tRNA). The fractionation markers, i.e. Tubulin (Cytoplasm), Lamin A/C (Nucleoplasm), Fibrillarin (Nucleoli), were tested to evaluate fractionation quality. (**d**) Images show localisation pattern of DNA (DAPI, Blue), a transfection FP-marker of pri-miR21-snoMEN (mCherry, Red) and the indicated proteins, i.e. Ago1, Ago2 and Upf1 (FITC, Green). Scale bar is 10 μm. Arrows and arrowheads show transfected and non-transfected cells, respectively. Specificity of each antibody was also verified by siRNA transfections (**Fig K** in S1 File). (**e**) HeLa cells were transfected with pri-miR21-snoMEN for 24 hours prior to fractionation. Cell lysates of each subcellular fraction were prepared and immunoprecipitated with either normal mouse IgG, or anti-Ago2 antibodies. Total protein was isolated from precipitates and resolved by SDS-PAGE and then analysed by western blotting using an Ago2 antibody to validate the efficiency of purification. In parallel, total RNA was also isolated from each precipitate and qRT-PCR was performed using the specific primers indicated.

We have previously demonstrated that the mechanism whereby snoMEN can modulate the expression level of a target mRNA requires Argonaute-2 (Ago2), which is in turn involved in the main RNAi pathway[32]. The snoMEN mechanism may also involve up-frameshift-1 (Upf1), which is thought to be essential to the nonsense-mediated decay (NMD) pathway[6, 33–35]. However, it was not clear whether snoMEN-mediated modulation of the expression level of a targeted non protein coding RNA, including pri-miRNAs, would involve exactly the same mechanism. Therefore, we examined whether RNAi-mediated depletion of several proteins, including Ago2, could affect the ability of snoMEN vectors to alter the expression of miRNAs (**Fig 7**). The results show that the snoMEN-dependent knock-down of pri-miR21 levels was prevented specifically in cells depleted of Ago2 by siRNA treatment, as compared with cells treated with a scrambled siRNA negative control (**Fig 7A and 7B)**. Additional controls showed that the level of snoMEN RNA expression wasn’t affected by siRNA transfections (**Fig J** in S1 File). This implies that Ago2 may be involved, either directly or indirectly, in the mechanism of snoMEN-mediated suppression of miRNA primary transcript levels. However, we found here that neither the siRNA-mediated depletion of Upf1, which was previously shown to affect the ability of snoMEN to knock-down protein coding RNAs[6], nor of Ago1, which, like Ago2[32], can associate with snoRNAs, prevented the inhibition of miR21 expression by snoMEN. These results suggest that Ago2, but not Upf1/Ago1, may potentially be involved in the mechanism through which snoMEN can reduce expression levels of miRNA primary transcripts.

To investigate the Ago2–snoMEN relationship further, the subcellular localisation of each of the Ago1, Ago2 and Upf1 proteins, was characterised using both cell fractionation and fluorescence microscopy analyses (**Fig 7C and 7D**). As judged by Western blotting, Ago1 and Upf1 are predominantly detected in the cytoplasmic fraction, with an additional weak signal in the nucleoplasm. In contrast, however, Ago2 was detected in cytoplasmic, nuclear and nucleolar fractions (**Fig 7C**). The results from this biochemical subcellular fractionation analysis were supported *in vivo* at the single cell level by fluorescence microscopy (**Fig 7D**). The localisation pattern of these Ago1, Ago2 and Upf1 proteins didn’t change after transfecting the pri-miR21-snoMEN vector, regardless of the level of transfection, as shown by the varying intensity of the mCherry transfection reporter in different cells (**Fig 7D**). The specificities of the antibodies used for imaging were confirmed by siRNA experiments (**Fig K** in S1 File). Nucleolar co-localisation was confirmed by co-staining with an antibody specific for the snoRNA-associated protein fibrillarin (**Fig L** in S1 File). These results are consistent with snoMEN interacting with Ago2 in the cell nucleus and nucleolus.

Next, the Ago2 protein was affinity-purified from each of the three subcellular fractions analysed above, which were isolated from cells transfected with snoMEN vectors. As a positive control, three known Ago2 associated snoRNAs (i.e. HBI-100, ACA45 and ACA25) [32, 36], were analysed by western blotting and qRT-PCR (**Fig 7E**). The purification of Ago2 protein from each subcellular fraction, i.e. Cytoplasm (**Fig 7E** lanes 1–4), Nucleoplasm (**Fig 7E** lanes 5–8) and Nucleoli (**Fig 7E** lanes 9–12), was validated by western blot analysis (**Fig 7E** Ago2). This showed that snoMEN RNA and all three of the known Ago2 interacting snoRNAs tested, i.e. HBI-100, ACA45, ACA25, co-purified with Ago2 proteins from both nuclear fractions, i.e. Nucleoplasm and Nucleoli. In contrast, neither of the negative control RNAs tested, i.e., U3 snoRNA and GAPDH mRNA, co-purified with Ago2 (**Fig 7E**). Taken together, these results are consistent with Ago2 associating with snoMEN RNAs and support the siRNA knock-down data indicating that Ago2 is required for the ability of snoMEN to reduce expression of targeted non-coding pri-miRNAs (**Fig M** in S1 File).

## Discussion

In this study we report the successful knock-down of miRNAs in human cells using snoMEN vectors targeted to miRNA primary transcripts. This extends our previous observations that the levels of protein coding mRNAs in human cells can be knocked-down upon expression of specific forms of genetically engineered nucleolar snoRNAs (i.e., snoMEN), which have the sequence of a short, internal region of the snoRNA altered to make it complementary to the RNA target. We show here that knock-down of miRNAs is lost if the engineered sequence in the snoRNA is mutated to reduce the degree of complementarity with the target, consistent with previous detailed studies showing that knock-down of pre-mRNA targets depends upon sequence complementarity in the snoMEN RNA[1]. While previous studies using snoMEN have exclusively made use of plasmid expression vectors for delivering snoMEN, here we have extended this to the expression of snoMEN RNAs from lentiviral vectors. This was shown to facilitate more efficient transduction of the snoMEN vectors into cultured cells and allowed the successful expression of snoMEN RNAs in cell lines that have proved difficult to transfect with conventional plasmid vectors. We also examined other types of primary human cells, e.g. skin fibroblast, breast epithelial and cortical neuronal cells. In each case the snoMEN lentiviral vectors showed successful transduction, resulting in mCherry expression in all cell types tested, consistent with previous studies[31] (data not shown). These results demonstrate that snoMEN RNAs can be delivered efficiently using lentivirus particles in multiple types of human cells, including both transformed and primary cells.

Interestingly, we observed that 4 snoMEN vectors targeted to different miRNAs showed ~2-fold higher knock-down efficiency of the respective targeted miRNAs, compared with the knock-down efficiency obtained with snoMEN targeted to miR21 (**Fig 4A**). We note that miR21 is known to be more abundant than the other miRNAs tested in HeLa cells[27]. This possibly explains why the knock-down efficiency observed with the snoMEN vectors varied, suggesting that it depends upon the copy number of the target miRNA.

Different micro RNAs have been found to play wide roles in regulating many biological response mechanisms. Hence the ability to modulate their expression via targeted knock-down strategies provides many potential applications for the analysis of gene regulation and conceivably also for the design of future therapeutic strategies. Here we have concentrated our analysis on modulating the expression of miR21, a micro RNA whose expression is highly up-regulated in many cancer cell lines and human tumours[8]. The data show that it is possible to reduce the expression level of mature miR21 using snoMEN vectors engineered to target complementary sequences in the miR21 precursor transcript. The level of miR21 knock-down achieved, while not complete, was sufficient to induce widespread apoptosis in transfected cells. The apoptosis phenotype appears to be directly related to the reduction of miR21 expression as it was not observed upon expression of multiple control snoMEN vectors that do not affect miR21 levels and was also suppressed when cells were transfected with the snoMEN miR21 knock-down vector in conjunction with an expression vector that boosted in parallel the total cellular levels of exogenous miR21. Furthermore, thanks to the development of lentiviral expression vectors for delivering snoMEN RNAs, we could show that expression of the mCherry–pri-miR21 snoMEN RNA, which is able to knock-down miR21 expression, did not induce apoptosis in human primary cells, which express low levels of endogenous miR21. However, when the exact same mCherry–pri-miR21 snoMEN RNA was transduced into human lung adenocarcinoma cells, which express high levels of miR21, this resulted in a strong cytotoxic phenotype and accompanying increase in multiple apoptotic markers within 3 days of transduction. Furthermore, preliminary proteomic analyses show that the expression levels of multiple proteins, including known downstream targets of miR21, such as KRAS and A/BRAF[37, 38], were down regulated specifically when lenti-mCherry-pri-miR21 snoMEN, but not the negative control lentiviral vectors, were transduced into cells (our unpublished observations).

Several previous reports describe knock-down of miRNAs by targeting either the short, mature miRNA sequences, or the pre-miRNA short hairpin molecules, using siRNA and/or shRNA[12, 13]. However, we are not aware of studies demonstrating the knock-down of miRNAs using siRNA or shRNAs targeted to the miRNA primary transcript, as shown here with the snoMEN vectors. Interestingly, while we confirmed the ability to inhibit miR21 with an shRNA vector targeting the mature miRNA sequence, neither siRNAs nor shRNA vectors targeted to the same pri-miR21 RNA sequences as expressed in the snoMEN vectors resulted in either effective miR21 knock-down, or induction of apoptosis. The use of siRNA/shRNA strategies, while often effective in reducing targeted RNA levels, has also been reported to cause indirect effects. For example the high level expression of exogenous siRNAs required for efficient gene silencing may cause saturation of the cellular miRNA maturation pathway, thereby altering expression levels of multiple endogenous miRNAs and thus altering cell growth and gene expression[14]. Our results to date, however, indicate that high level expression of exogenous snoMEN RNAs does not prevent or reduce cell growth, or alter the expression profile of a wide range of genes, as measured by a quantitative MS approach[1, 6]. This is likely because endogenous box C/D snoRNAs are highly abundant nuclear RNAs that are very efficiently processed from within introns of many different protein-coding cellular pre-mRNAs. It is therefore unlikely that exogenous expression of the M box-modified snoMEN RNAs will alter endogenous snoRNA expression.

Interestingly, our data suggest that the mechanism of miRNA knock-down by snoMEN requires Ago2, but not Ago1 or Upf1. Co-immunoprecipitation studies show that Ago2 is associated with snoMEN RNA in both nucleoplasmic and nucleolar extracts (**Fig 7**). While our data confirm previous reports that high levels of Ago2 localise to the cytoplasm, where it functions in the RNA interference pathway[14–16, 39] (**Fig 7**and **Figs K** and **L** in S1 File), spatial proteomic analysis of human cell extracts show that a significant proportion (~30–40%) of Ago2 also localises in the nucleus[40, 41]. In addition, reports of nuclear functions for Ago2 are currently expanding[42–44]. A subset of miRNAs, including miR21, temporarily localise in the nucleolus during their maturation [45, 46]. Our previous studies on snoMEN targeting of protein coding mRNAs indicated that this involved an antisense pathway that occurred mainly in the nucleus and involved both Ago1 and Upf1 proteins. Taken together, the present data suggest that pri-miRNA targeting by snoMEN also involves antisense RNA base pairing in the nucleus but primarily requires Ago2 and not Upf1. It will be interesting in future to characterise in greater detail how Ago2 may be involved in the mechanism of action of snoMEN RNAs. We anticipate that it may also be possible to improve further the efficiency of knock-down by snoMEN RNAs when the vector design and mechanism of action is understood in more detail. Given the successful demonstration here of how snoMEN can specifically induce apoptosis in human cancer cells, further development of snoMEN vectors may thus offer a useful new approach for anti-tumour therapy that can complement existing strategies.

## Methods

### Cell culture and plasmid transfection

The human cervical cancer HeLa cell line and human embryonic kidney derived 293T cell line were provided from EMBL and ATCC, respectively. HeLa cells and 293T cells were maintained in Dulbecco’s modified Eagle’s medium (DMEM) supplemented with 10% fetal bovine serum (FBS). All plasmid transfections were performed using Effectin (QIAGEN), as described by the supplier. ATCC-CCL-75 (passage 16) human lung fibroblast and ATCC-CRL-5868, human lung adenocarcinoma cells were purchased from ATCC and maintained in either DMEM, or RPMI 1640, supplemented with 10% fetal bovine serum (FBS).

### Plasmid construction

SnoMEN vectors were established as previously described[1, 6]. The sequence spanning exon 2 to exon 3 of the C19orf48 gene was inserted 5′ of the EGFP-/mCherry–N1 mammalian expression plasmid (Clontech) (**Fig 1A**). The M box sequence of HBII-180C cDNA (5′-CACCCCTGAGGACACAGTGCA-3′) was modified to create complementary sequences to target genes as follows; pri-miR21: set1 5’-TGGATGGTCAGATGAAAGATACC-3’, set2 5’-TACCCGACAAGGTGGTACAGCCA-3’, set3 5’-GCCATGAGATTCAACAGTCAA-3’, pre-miR21: set4 5′-ACCAACGGTCTGGTAAAGAGT-3′, set5 5′-GGATCAGTTACCTCATTAGAA-3′ and set6 5′-CCCACCTTAACTCTCCTCCCC-3′. Control (snoMEN targeted to EGFP[1, 6]): CM1 5’-GACTTGAAGAAGTCGTGCTGC-3’, CM2 5’-ACCTTGATGCCGTTCTTCTGC-3’, CM3 5’-ATGATATAGACGTTGTGGCTG-3’. snoMEN constructs were also subcloned into the Lenti-X expression system (Clontech). snoMEN lentivirus particles were produced and transduced into cells as described by the supplier (see also below).

### Fluorescent *in situ* hybridization (FISH)

The FISH procedure was performed as previously described [http://www.singerlab.org/protocols][1, 6]. HeLa cells were transfected with a plasmid vector that expressed the snoMEN RNA from the CMV promoter. The cells were fixed with 4% paraformaldehyde after pre-permeabilization with 1% tritonX-100. After 70% ethanol treatment, Cy-3 labeled RNA oligonucleotide probes, complementary to each of the M box specific sequences, were hybridized using standard procedures. The fluorescence signal was imaged using a Deltavision Spectris fluorescence microscope (Applied Precision).

### Microscopy and antibodies

All cell images were recorded using the DeltaVision Spectris fluorescence microscope (Applied Precision). Cells were imaged using either a 10X, or 60X (NA 1.4), Plan Apochromat objective lens. Twelve optical sections, each separated by 0.5 μm, were recorded for each field and each exposure (SoftWoRx image processing software, Applied Precision). Primary antibodies against Fibrillarin (72b9)[47], Tubulin (DM1A, Sigma), Cleaved Caspase-3 (Asp175, Cell Signalling Technology), Cleaved PARP (Asp214, Cell Signalling Technology), SMN1 (BD Transduction Laboratories), Ago1 (D84G10, Cell Signalling Technology), Ago2 (C34C6, Cell Signalling Technology), Upf1 (Cell Signalling Technology) and Lamin A/C (636 Santa Cruz), were used for immunostaining and/or western blotting.

### RNA isolation and quantitative PCR/RT-PCR

Total RNA was isolated by the TRIzol method with DNase I treatment (Invitrogen) and converted to cDNA using the qScript microRNA cDNA Synthesis kit, according to the manufacturer’s instructions (Quanta Biosciences). qRT-PCR and qPCR reactions were performed to detect miRNA precursor (pre-miRNAs), and matured miRNAs, respectively. The QuantiFast SYBR green RT-PCR kit (QIAGEN) and PerfeCta SYBR Green qPCR kit (Quanta Biosciences), were used to analyse samples for qRT-PCR and qPCR, respectively, on the LightCycler 480 II platform (Roche). U3 snoRNA and/or GAPDH mRNA was used as a normalisation control for RNA expression levels in all experiments. PCR primer sequences are listed in supplementary information. Each experiment was repeated at least three times independently.

### Northern RNA blot analysis

Total cell RNA was isolated using the TRIzol method, with DNase I treatment, according to the manufacturer’s instructions (Invitrogen). Equal amounts of RNA from each sample were separated by 8M Urea polyacrylamide denaturing gel electrophoresis in 1xTBE buffer and the RNA transferred onto nylon membrane (Hybond-N; Amersham) by electro blotting. After either UV cross linking, or chemical cross linking[48], the membrane was hybridized with ^32^P 5’ end-labelled oligoribonucleotide probes specific for the following RNA species; (miR21: 5’-UCAACAUCAGUCUGAUAAGCUA-3’, tRNA-Ile: 5’-UGGUGGCCCGUACGGGGAUCGA-3’, snoMEN: 5’-CCCCAGGUGUCAAUUUUCCUGUUU-3’).

### Production and transduction of Lentivirus

snoMEN constructs were subcloned into the Lenti-X expression system (Clontech) (**Fig 4A**). VSV-G pseudo typed lentiviruses were generated by transient co-transfection of 293T cells with a three-plasmid combination as follows: One T75 flask containing 1x10^7^ 293T cells was transfected using the effectine transfection reagent (QIAGEN) with 5 μg lentiviral vector, 3.75 μg pCMV Δ8.91 and 1.25 μg pMD VSV-G. Supernatants were collected 72 hr after transfection, pooled together and concentrated using the Lenti-X Maxi purification kit (Clontech). Virus titration was measured by Lenti-X GoStix / Lenti-X qRT-PCR Titration kits, according to the manufacturer’s instructions (Clontech). The purified viruses were stored frozen at -80°C. For lentiviral transduction, 1x10^5^ cells/well were seeded in 6 well tissue culture plates and infected the following day with lentiviruses encoding the respective snoMEN RNAs as described in the Results section.

### shRNA experiments

Plasmids vectors expressing shRNAs were constructed using the pLVX-shRNA2 vector, as described by the supplier (Clontech). Control shRNA (EGFP: 5’- ACCTTGATGCCGTTCTTCT-3’) and miR21 shRNA (sh21: 5’-CATCAGTCTGATAAGCTAC-3’) were transfected as positive and negative controls, respectively. M box shRNA sequences are as follows; miR21 Mbox shRNA-1 (sh21M1): 5′-TGGTCAGATGAAAGATACC-3′, miR21 Mbox siRNA-2 (sh21M2): 5′-CCCGACAAGGTGGTACAGC-3′, miR21 Mbox siRNA-3 (sh21M3): 5′-TGGTCAGATGAAAGATACC-3′.

### siRNA experiments

siRNA was transfected by Lipofectamine RNAiMAX (Invitrogen) according to the manufacturer’s instructions. Control ‘scrambled’ siRNA, which does not have an endogenous RNA target (Dharmacon), and miR21 siRNA (si21: 5′-ACAUCAGUCUGAUAAGCUACC-3′) (siMAX siRNA, MWG operon), were transfected as negative and positive controls, respectively. The miR21 M box siRNAs, targeted to the same precursor sequences as used for the pri-miR21 snoMEN, were synthesised and transfected (miR21 Mbox siRNA-1 (si21M1): 5′-UGGAUGGUCAGAUGAAAGAUACC-3′, miR21 Mbox siRNA-2 (si21M2): 5′-UACCCGACAAGGUGGUACAGCCA-3′, miR21 Mbox siRNA-2 (si21M3): 5′-ACGAUGGUAGGCAAAACAAGCAG-3′, (siMAX siRNA, MWG operon)). Upf1/Rent1, Argonaute-1 (Ago1)/EIF2C1, Argonaute-2 (Ago2)/EIF2C2 siRNAs (On-Targetplus SMART pool product, Dharmacon) were also transfected into Hela cells using Lipofectamine RNAiMAX[6].

### Cell fractionation and Immunoprecipitation

Cytoplasm, nuclei and nucleoli were prepared from HeLa cells essentially as previously described[1, 41, 49–51]. Briefly, cells were washed three times with PBS, resuspended in 5 ml buffer A (10 mM HEPES- KOH [pH 7.9], 1.5 mM MgCl2, 10 mM KCl, 0.5 mM DTT), and dounce homogenized ten times using a tight pestle. Dounced nuclei were centrifuged at 228 × *g* for 5 min at 4°C. The supernatant represents the cytoplasmic fraction. The nuclear pellet was resuspended in 3 ml 0.25 M sucrose, 10 mM MgCl2, and layered over 3 ml 0.35 M sucrose, 0.5 mM MgCl2, and centrifuged at 1430 × *g* for 5 min at 4°C. The clean, pelleted nuclei were resuspended in 3 ml 0.35 M sucrose, 0.5 mM MgCl2 and sonicated for 6 × 10 s using a microtip probe and a Misonix XL 2020 sonicator at power setting 5. The sonication was checked using phase contrast microscopy, ensuring that there were no intact cells and that the nucleoli were readily observed as dense, refractile bodies. The sonicated sample was then layered over 3 ml 0.88 M sucrose, 0.5 mM MgCl2 and centrifuged at 2800 × *g* for 10 min at 4°C. The pellet contained the nucleoli, while the supernatant consisted of the nucleoplasmic fraction. The nucleoli were then washed by resuspension in 500 μl of 0.35 M sucrose, 0.5 mM MgCl2, followed by centrifugation at 2000 × *g* for 2 min at 4°C.

Immunoprecipitations were prepared as previously described**[52, 53]**. Nuclear lysates were prepared from HeLa cells that had been transfected with the pri-miR21snoMEN expression plasmid. Purified nuclei were resuspended in RIPA buffer to solubilize proteins. Ago2 proteins were immunoprecipitated using an anti-Ago2 monoclonal antibody (Cell Signalling Technology) covalently coupled to protein G-Sepharose, as previously described. Samples were divided in two and for Input samples RNA was isolated from one half of each lysate. RNA was isolated by the TRIzol method with DNase I treatment, according to the manufacturer’s instructions (Invitrogen). RT-PCR was performed to detect immunoprecipitated RNAs. PCR primer sequences are listed in supplementary information.

## Supporting Information

S1 File Supporting information. (PDF)
