# Supplementary material for: Targeted Knock-Down of miR21 Primary Transcripts Using snoMEN Vectors Induces Apoptosis in Human Cancer Cell Lines
Source: PLoS One. 2015 Sep 25;10(9):e0138668. doi: 10.1371/journal.pone.0138668 (PMC4583369; doi:10.1371/journal.pone.0138668)
Supplement: S1 File — (PDF) [file pone.0138668.s001.pdf]

Supporting Information

Supporting Information Captions

S1 File. Supporting information of the study "Targeted knock-down of miR21 primary transcripts using snoMEN vectors induces apoptosis in human cancer cell lines". miR21 knock-down by snoMEN vector targeted to miR21 precursor sequence (**Figures A-D**). Expression of snoMEN molecules delivered using lentiviral vectors (**Figures E** and **F**). snoMEN knock-down using lentiviral vectors (**Figures G** and **H**). Pri-miR21 targeted shRNAs do not induce apoptosis (**Figure I**)**.** Ago2 is important for snoMEN RNA knock-down of miRNA (**Figures J-L**). Schematic diagram depicting a model for the snoMEN RNA interference pathway (**Figure M**). Primer sequences (**Primer sequences**)

A Fig. Structure for targeted endogenous miR21 precursor sequence (mCherry–pre-miR21 snoMEN) and schematic diagram of miR21 primary transcript. This construct has three snoMEN sequences (orange pentagons) as previously described[1], except that here the M box sequences are complementary to specific sequences in the endogenous miR21 precursor sequence of the primary transcript.

B Fig. Validation of snoMEN RNA expression by Fluorescence In Situ Hybridisation (FISH) analysis. snoMEN expression was detected by using a M box specific RNA probe labelled with Cy3 (Cy3). DNA is stained by DAPI (DAPI). Scale bar is 10 µm. Arrow shows nucleolus.

C Fig. FACS analysis. Graph shows a time course of changes in the population of Annexin-V positive cells transfected with either pre-miR21-snoMEN (Green), or Control (Red). The cell number was counted using FACS. Annexin-V signal was detected by using Guava Nexin Reagent (Guava technologies).

D Fig. RNA analysis. Total RNA from HeLa cells was harvested 24 hours after transfection and qRT-PCR was performed to identify miR21 precursor molecules using miR21 precursor specific primers (pre-miR21). Following cDNA synthesis, qPCR was performed using matured miR21 specific primers and universal primers provided by the PerfeCta SYBR Green qPCR kit (Quanta Biosciences, see also methods) (Matured-miR21). U3 snoRNA was used as a control. Graph depicts mean and standard deviation from a minimum of 5 independent experiments.

E Fig. Validation of snoMEN expression by Fluorescence In Situ Hybridisation (FISH) analysis. Each snoMEN RNA was detected using a M box specific RNA probe labelled with Cy3 (Cy3). DNA is stained by DAPI (DAPI). Scale bar is 10 μm. Arrows show nucleoli.

F Fig. RNA analysis. Total RNA from human lung primary cells (ATCC-CCL-75) and lung cancer cells (ATCC-CRL-5868) was harvested. Following cDNA synthesis, qPCR was performed using matured miR21 specific primers and universal primers provided by the PerfeCta SYBR Green qPCR kit (Quanta Biosciences, see also methods). GAPDH was used as a control. Graph depicts mean and standard deviation from a minimum of 4 independent experiments.

G Fig. Micrograph show apoptosis induction following transfection with Lenti-mCherry–pre-miR21-snoMEN. Images were taken 72 hours after transfection. Scale bar is 10 μm.

H Fig. RNA analysis. Total RNA from lung primary (ATCC-CCL-75) and lung cancer cells (ATCC-CRL-5868) was harvested 24 hours after transduction. Following cDNA synthesis, qPCR was performed using matured miR21 specific primers and universal primers provided by the PerfeCta SYBR Green qPCR kit (Quanta Biosciences, see also methods). GAPDH was used as a control. Graph depicts mean and standard deviation from a minimum of 4 independent experiments.

I Fig. Micrographs show cell phenotypes after transfecting with shRNA targeting to pri-miR21. Images were taken 72 hours after transfection. Green signals indicate the expression of the zs green transfection marker protein (c.f. vector diagram in manuscript Figure 6). Arrows indicate cells showing an apoptosis phenotype. Scale bar is 10 μm.

J Fig. HeLa cells were transfected with siRNA oligonucleotides prior to pri-miR21-snoMEN transfection for 24h. Total RNA from HeLa cells was harvested and qPCR was performed, following cDNA synthesis, using snoMEN specific primers and universal primers provided by the PerfeCta SYBR Green qPCR kit (Quanta Biosciences, see also methods).

K Fig. HeLa cells were transfected with siRNA oligonucleotides, targeted to the indicated genes, for 48 hours. Images show localisation pattern of DNA (DAPI, Blue) and the indicated proteins, i.e. Ago1, Ago2 and Upf1 (FITC, Green). Scale bar is 10 μm.

L Fig. Images show localisation pattern of DNA (DAPI, Blue), Ago2 (Red) and a nucleolar marker protein (Fibrillarin, Green). Scale bar is 10 μm. Arrows show co-localisation in nucleoli between Ago2 and Fibrillarin.

M Fig. Schematic diagram depicting a model for the snoMEN RNA interference pathway. snoMEN RNP complexes modulate gene expression by targeting either pre-mRNAs or pri-miRNAs in the nucleus. The snoMEN RNA complexes induce RNA degradation, which requires Ago2 and/or Upf1 in the case of pre-mRNAs, or specifically requires Ago2 in the case of pri-miRNAs. snoMEN may target pri-miRNAs either in or out of the nucleolus.

Primer sequences

Pre-miR21: 5'-TGTCGGGTAGCTTATCAGACT-3' and 5'-TGTCAGACAGCCCATCGACTGG-3'.

Matured-miR21: 5'-TAGCTTATCAGACTGATGTTGA-3' and Universal primer (Quanta Biosciences).

SnoMEN: 5'-ACCTTGATGCCGTTCTTCTGC-3' and Universal primer (Quanta Biosciences).

U3: 5'-AGAGGTAGCGTTTTCTCCTGAGCG-3' and 5'-ACCACTCAGACCGCGTTCTC-3'.

HBI-100: 5'-TATGGAGGTCTCTGTCTGGCTT-3' and 5'-TGTACGGAGGGGAACGATCAGA-3'.

ACA45: 5'-AAGGTAGATAGAACAGGTCTTG-3' and 5'-TGCTGTTGGTAGATAAGTAGG-3'.

ACA25: 5'-GTTCTCTATAGGAAGCCATAGC-3' and 5'-TTTTATATCTCCTCAGGAAAACATAG-3'.

GAPDH: 5'-TTGCGTCGCCAGCCGAGCCACATC-3' and 5'-CAATACGACCAAATCCGTTGACTCCGA-3'.

Let-7g: 5'-TGAGGTAGTAGTTTGTACAGTT-3' and Universal primer (Quanta Biosciences).

miR132: 5'-TAACAGTCTACAGCCATGGTCG-3' and Universal primer (Quanta Biosciences).

miR31: 5'-AGGCAAGATGCTGGCATAGCT-3' and Universal primer (Quanta Biosciences).

miR210: 5'-CTGTGCGTGTGACAGCGGCTGA-3' and Universal primer (Quanta Biosciences).

Fig A

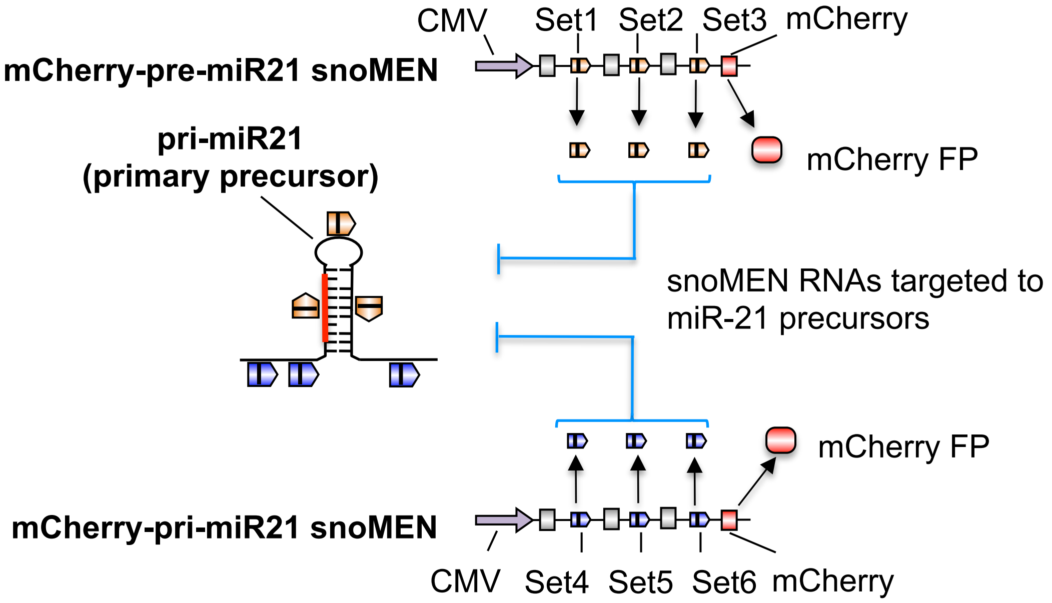

Fig B

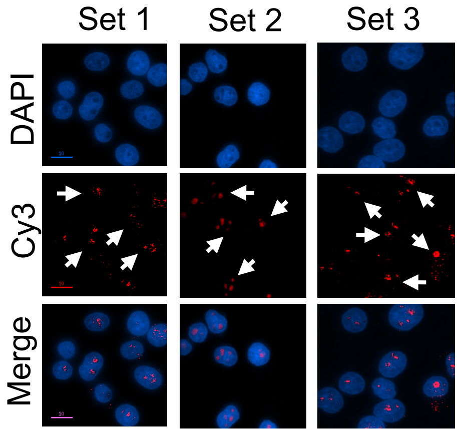

Fig C

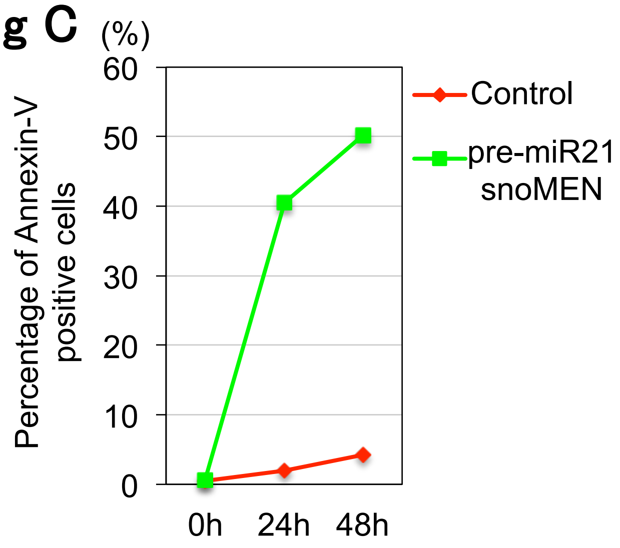

Fig D

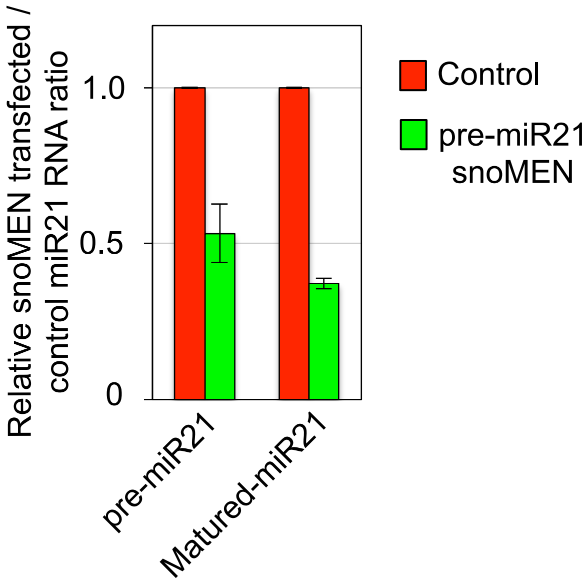

Fig E

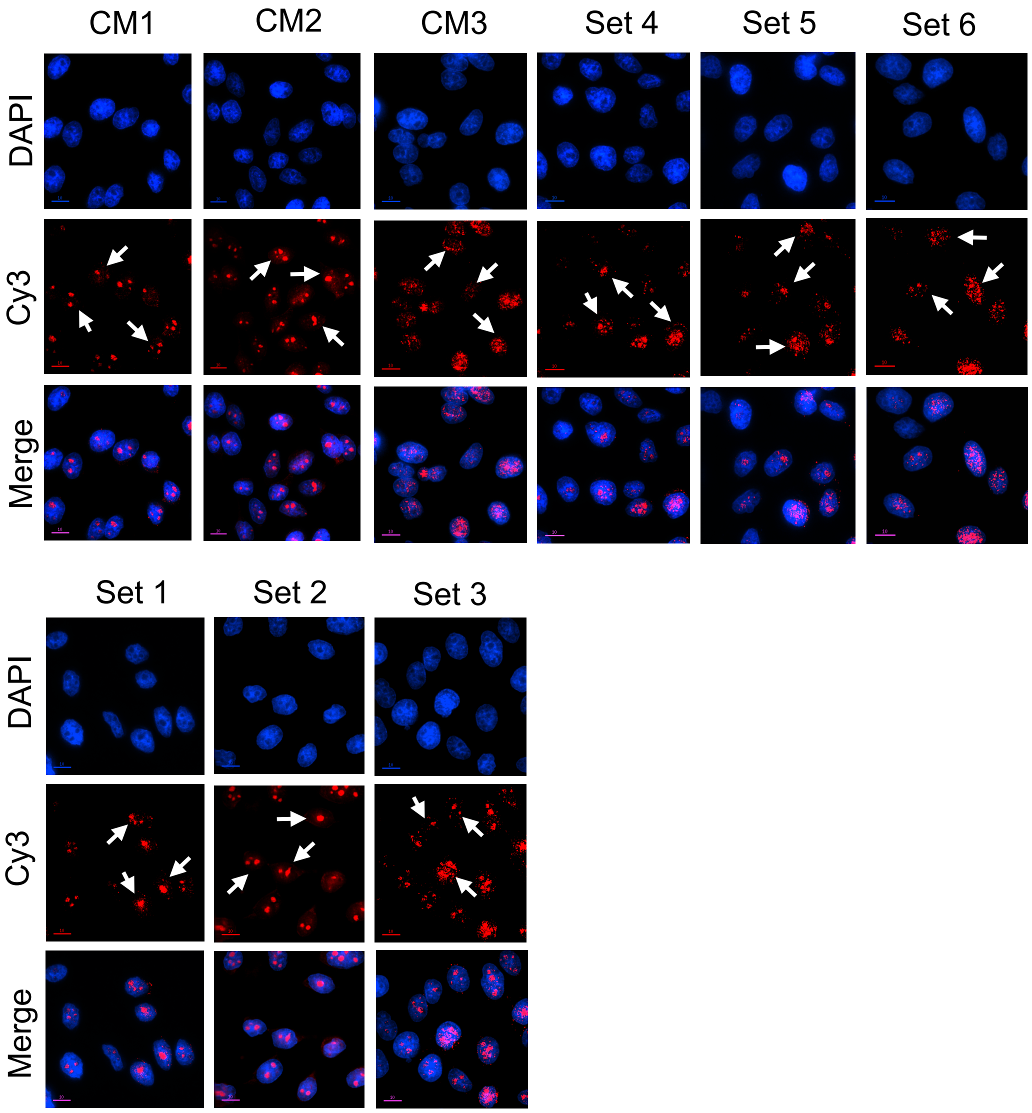

CM1
CM2
CM3
Set 4
Set 5
Set 6
DAPI
Cy3
Merge
Set 1
Set 2
Set 3
DAPI
Cy3
Merge

Fig F

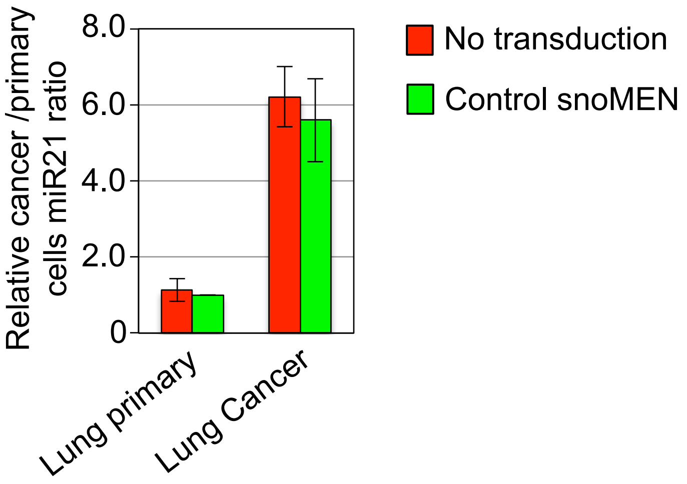

Relative cancer /primary cells miR21 ratio
8.0
6.0
4.0
2.0
0
Lung primary
Lung Cancer
No transduction
Control snoMEN

Fig G

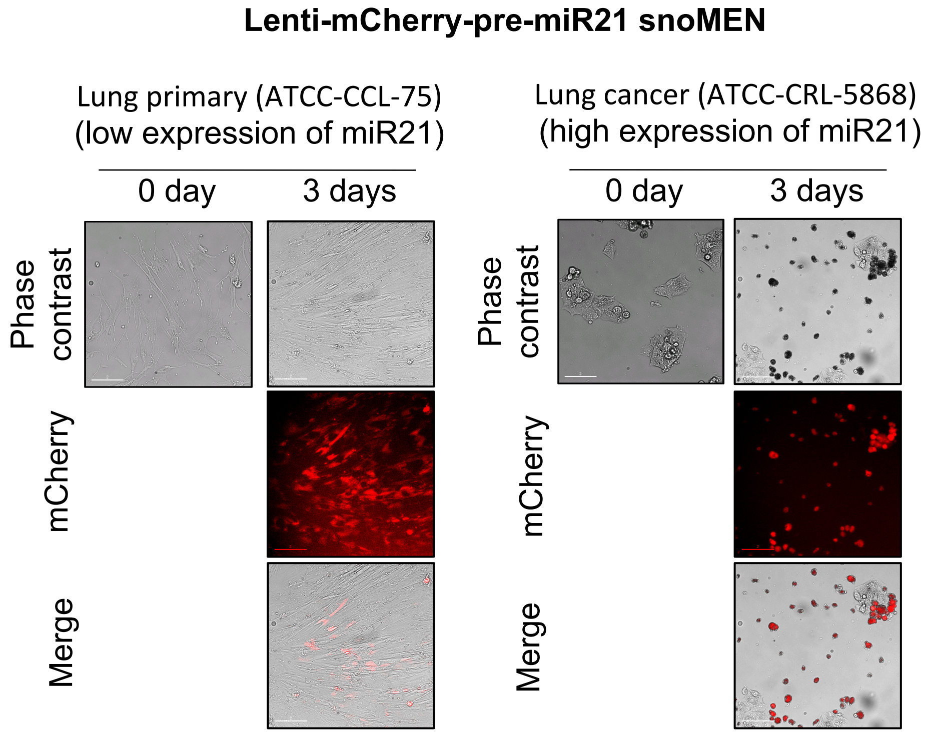
Lenti-mCherry-pre-miR21 snoMEN
Lung primary (ATCC-CCL-75)
(low expression of miR21)
Lung cancer (ATCC-CRL-5868)
(high expression of miR21)
0 day
3 days
0 day
3 days
Phase contrast
mCherry
Merge
Phase contrast
mCherry
Merge

Fig H

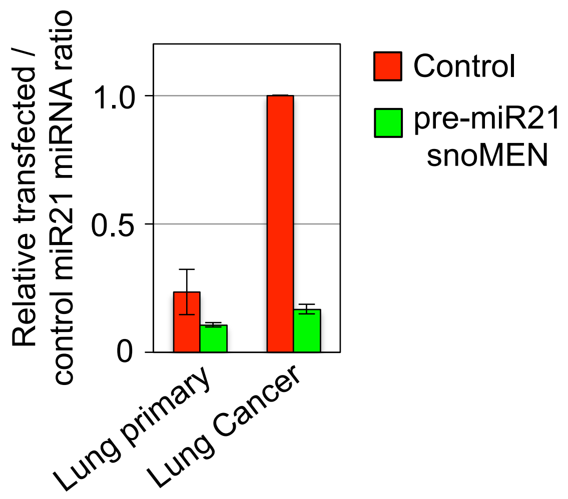
Relative transfected / control miR21 miRNA ratio
1.0
0.5
0
Lung primary
Lung Cancer
Control
pre-miR21 snoMEN

Fig I

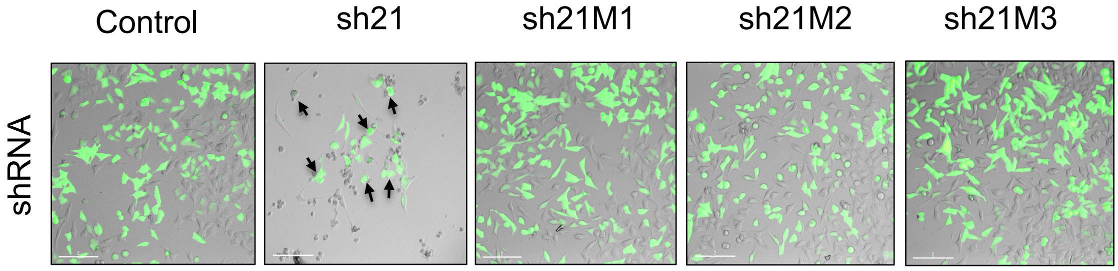

Fig J

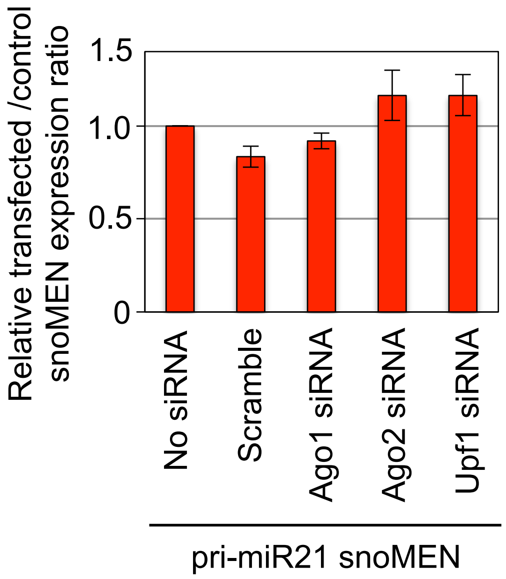

Fig K

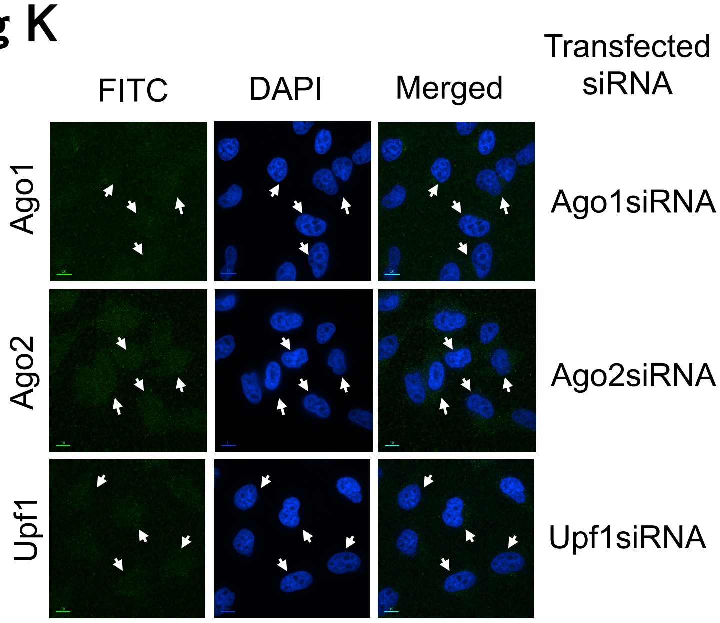

Fig L

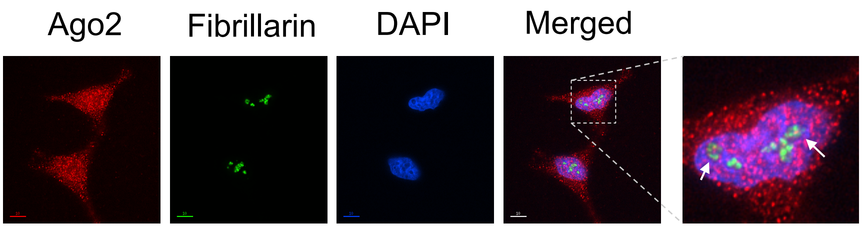
Ago2
Fibrillarin
DAPI
Merged

Fig M

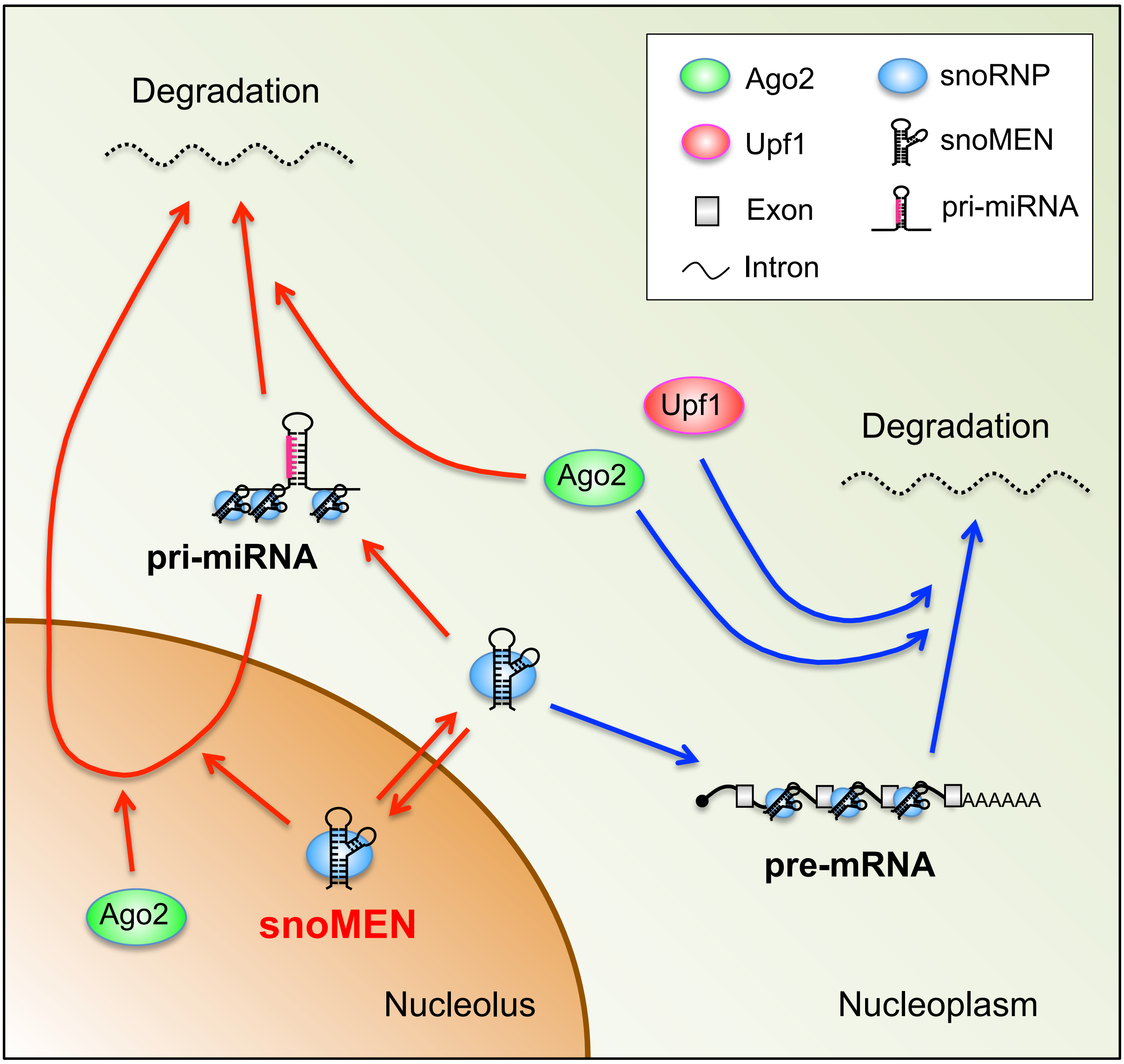
Degradation
Ago2
snoRNP
Upf1
snoMEN
Exon
pri-miRNA
Intron
Upf1
Degradation
Ago2
pri-miRNA
AAAAAA
pre-mRNA
Ago2
snoMEN
Nucleolus
Nucleoplasm
